# Editorial: Non-coding RNAs and Graft versus Host Disease

**DOI:** 10.3389/fimmu.2018.02713

**Published:** 2018-11-20

**Authors:** Pavan Reddy, Robert Zeiser

**Affiliations:** ^1^Department of Internal Medicine, University of Michigan Comprehensive Cancer Center, Ann Arbor, MI, United States; ^2^Department of Hematology, Oncology and Stem Cell Transplantation, Freiburg University Medical Center, Albert Ludwigs University of Freiburg, Freiburg, Germany

**Keywords:** GvHD, micro RNA, Inflammation, non-coding RNAs, T cell

Acute graft-vs.-host disease (GVHD) causes activation of multiple pro-inflammatory pathways in a diverse array of immune cell subsets that are often regulated by micro RNAs (miRs). MiRs are small non-coding RNAs that post-transcriptionally regulate gene expression and thereby impact inflammatory events at multiple levels including the release cytokines, chemokines, signaling by their receptors, cell-cycling, migration, and filament stabilization. Multiple inflammation-related miR-target genes are described, and while some miRs target anti-inflammatory genes other promote inflammation by targeting immunosuppressive genes. Additionally, the same miR can have different effects by preferentially targeting certain genes depending on the cell type and their context, in which the miR is analyzed. Through these diverse effects in various critical immune cell subsets miRs play critical functional role in GVHD and graft-vs.-leukemia effects (GVL). Thus, miRs are potentially attractive targets for the modification of allogeneic immune responses using miR mimics and inhibitors. Additionally, miR-levels in different body fluids could help to guide clinical decision making and function as biomarkers to predict or diagnose GVHD. This Research Topic, authored by experts in the field, describes the pleomorphic function of different miRs as potent regulators of multiple pro- or anti-inflammatory target genes, in various immune cell subsets that affect alloimmunity and GVHD. A better understanding of the miR-biology may help to mitigate GVHD and augment GVL effects and improve the outcomes after allogeneic hematopoietic transplantation.

## Author contributions

All authors listed have made a substantial, direct and intellectual contribution to the work, and approved it for publication.

### Conflict of interest statement

The authors declare that the research was conducted in the absence of any commercial or financial relationships that could be construed as a potential conflict of interest.

